# Pleural Fluid Biomarkers in Focus: The Role of Cholesterol and Lactate Dehydrogenase (LDH) in Differentiating Exudative From Transudative Effusions

**DOI:** 10.7759/cureus.87811

**Published:** 2025-07-13

**Authors:** AkhilAnand PG, Elen Ann Abraham, Ghanshyam Verma, Keerthana Prakash, Pedada Mounika

**Affiliations:** 1 Department of Respiratory Medicine, Sree Balaji Medical College and Hospital, Chennai, IND

**Keywords:** biological markers, diagnostic techniques and procedures, exudative, pleural effusion, pleural fluid cholesterol, pleural fluid lactate dehydrogenase, transudative

## Abstract

Background

Pleural effusion affects approximately 320 individuals per 100,000 population and can result from a wide range of pulmonary and extrapulmonary conditions. It may present as a complication of an existing illness or as an initial clinical sign, making accurate diagnosis and timely management essential. Under normal physiological conditions, the pleural space contains 7-16 mL of fluid (approximately 0.26 ± 0.1 mL/kg in healthy non-smokers). Effusion develops when fluid production exceeds clearance. Based on pathophysiology, pleural effusions are classified as either transudates or exudates. Light’s criteria remain the standard diagnostic tool but may misclassify up to 25% of cases. Recent studies suggest that pleural fluid cholesterol (pCHOL) may offer superior diagnostic accuracy.

Aim

This study aims to evaluate the reliability and diagnostic accuracy of pCHOL and lactate dehydrogenase (LDH) in distinguishing between transudative and exudative effusions in a tertiary care setting.

Methods

In this cross-sectional study, 80 patients with pleural effusion were classified as transudative (n = 30) or exudative (n = 50) based on clinical and radiological criteria. Paired pleural fluid and serum samples were collected. Pleural fluid protein, LDH, and cholesterol levels, as well as serum protein and LDH, were analyzed to apply Light’s criteria. Diagnostic performance was evaluated using sensitivity, specificity, and predictive values. Statistical analysis was conducted using IBM SPSS Statistics for Windows, Version 21.0 (Released 2012; IBM Corp., Armonk, NY, US), with p < 0.05 considered statistically significant.

Results

Mean pCHOL was significantly higher in exudative effusions (68.5 ± 20.3 mg/dL) than in transudative effusions (25.2 ± 9.6 mg/dL, p < 0.001). At a cutoff value of 45 mg/dL, pCHOL showed a sensitivity of 98% and specificity of 100%. This outperformed the pleural/serum protein ratio (78%/82%), pleural/serum LDH ratio (86%/93%), and pleural LDH level (88%/90%). The statistically significant difference in pCHOL levels underscores its superior diagnostic utility (p < 0.001).

Conclusion

pCHOL is a highly sensitive and specific biomarker for identifying exudative pleural effusions and demonstrates better diagnostic performance than Light’s criteria. Its simplicity, cost-effectiveness, and diagnostic reliability support its routine inclusion in pleural fluid analysis.

## Introduction

Pleural effusion, defined as the pathological accumulation of fluid in the pleural space, remains a common diagnostic challenge in clinical practice [1,2]. Accurate differentiation between exudative and transudative effusions is critical, as it guides further evaluation and management strategies. Light’s criteria have long served as the standard for this classification [3], but they are not without limitations, particularly in cases involving patients on diuretics, where misclassification can occur [4].

Emerging evidence indicates that pleural fluid cholesterol (pCHOL) and lactate dehydrogenase (LDH) may serve as reliable and cost-effective biomarkers for differentiating exudative from transudative effusions [5-7]. Several studies have shown that pCHOL provides diagnostic accuracy comparable to, or even exceeding, that of Light’s criteria, especially in challenging scenarios such as diuretic-treated heart failure or parapneumonic effusions [8-10]. However, most existing data are derived from Western populations, and there is limited evidence from the Indian subcontinent, where the spectrum of etiologies and healthcare resource constraints necessitate local validation [11,12]. This study was conducted at a tertiary care hospital in India to evaluate the diagnostic performance of pCHOL and LDH. It aimed to assess their clinical utility in real-world settings and to explore their potential as accessible, cost-effective alternatives to Light’s criteria. Specifically, the study examined the diagnostic reliability of pCHOL and LDH in distinguishing exudative from transudative pleural effusions and compared their performance with the individual components of Light’s criteria.

## Materials and methods

This prospective observational study was conducted over six months, from October 2022 to March 2023, in the Department of Respiratory Medicine at Sree Balaji Medical College and Hospital, Chennai. The study protocol was approved by the Institutional Human Ethics Committee (Ref. No. 002/SBMCH/IHEC/2022/1795, dated September 22, 2022).

Study population

Consecutive adult patients (aged ≥18 years) with clinically and radiologically confirmed pleural effusion were enrolled after obtaining written informed consent. Exclusion criteria included patients under 18 years of age, those who declined to provide consent, individuals lacking a definitive clinical or radiological diagnosis of pleural effusion, and patients diagnosed with pulmonary embolism.

**Figure 1:**
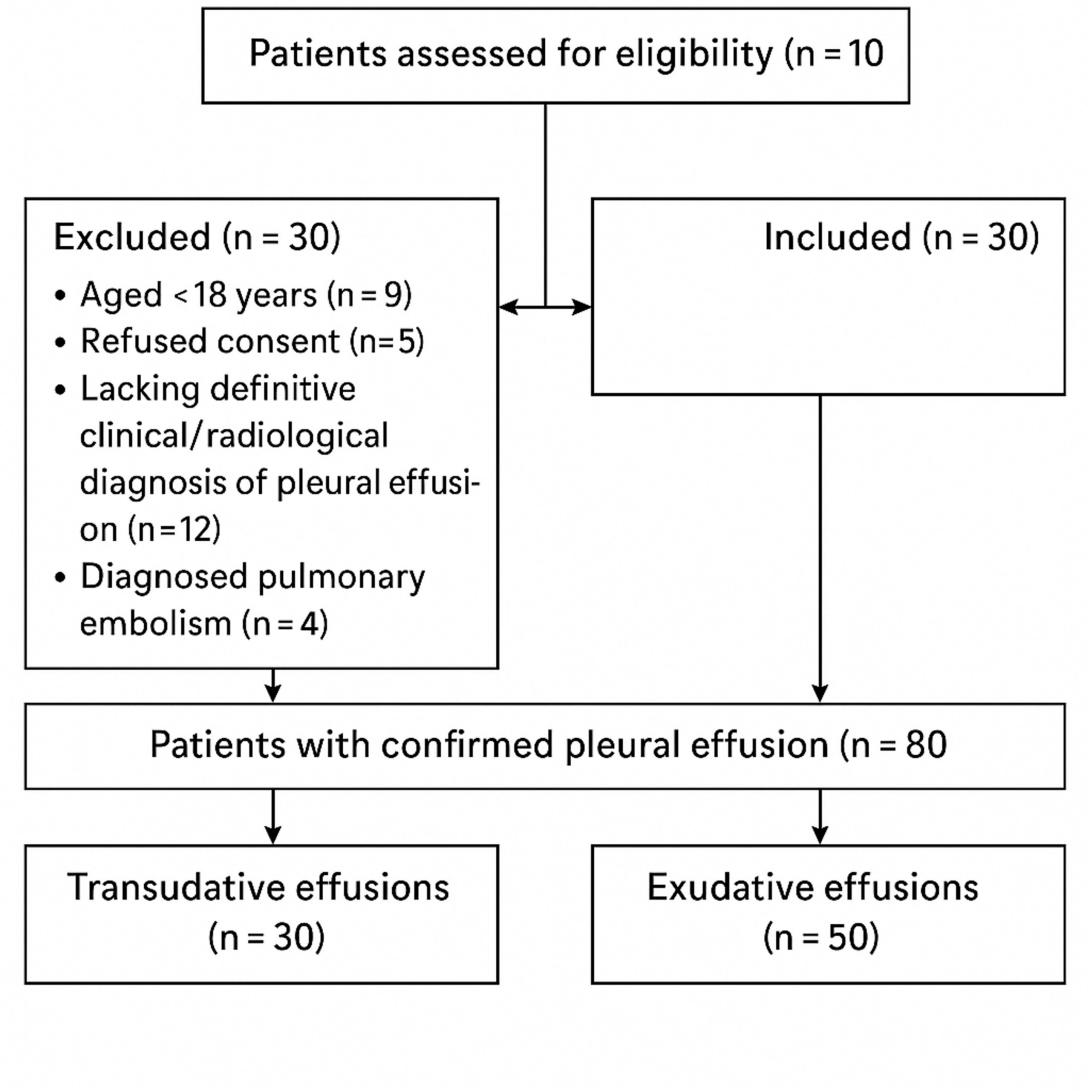
Flowchart of patient recruitment and classification A total of 110 patients with suspected pleural effusion were assessed for eligibility. Thirty patients were excluded due to age below 18 years (n = 9), refusal to consent (n = 5), lack of definitive clinical or radiological diagnosis (n = 12), or confirmed pulmonary embolism (n = 4). Eighty patients were included in the study, comprising 50 cases of exudative pleural effusion and 30 cases of transudative pleural effusion.

Figure 1 illustrates the patient recruitment, exclusions, and allocation into transudative and exudative groups.

Classification of effusions

Effusions were classified as transudative, typically associated with systemic conditions such as congestive heart failure, hepatic cirrhosis, or nephrotic syndrome, or exudative, which are linked to local inflammatory or pathological processes such as pneumonia, tuberculosis, malignancy, or other pleuroparenchymal infections. Classification was based on Light’s criteria and validated using clinical and radiological findings.

Pleural fluid and serum analysis

Pleural fluid samples were collected under aseptic precautions during diagnostic thoracentesis. Simultaneously, venous blood samples were obtained for serum analysis. All specimens were transported to the laboratory within 30 minutes of collection and processed promptly.

Pleural fluid was subjected to routine biochemical analysis, including measurements of total protein, LDH, and cholesterol levels. pCHOL was measured using the enzymatic cholesterol oxidase-phenol aminoantipyrine (CHOD-PAP) method.

All biochemical assays were performed using standardized, automated analyzers (e.g., Beckman Coulter AU5800, Beckman Coulter, Brea, CA, US) in accordance with internal quality control protocols. Laboratory reference ranges were as follows: serum LDH 140-280 IU/L and serum cholesterol 125-200 mg/dL.

Corresponding serum samples were analyzed for protein and LDH concentrations. The following diagnostic parameters were calculated: pleural/serum protein ratio, pleural/serum LDH ratio, and absolute pleural fluid LDH and cholesterol concentrations.

Effusions were classified as exudative based on established criteria [13]: pleural/serum protein ratio > 0.5; pleural/serum LDH ratio > 0.6; pleural fluid LDH > two-thirds of the upper limit of normal (ULN) serum LDH; and pleural fluid cholesterol > 45 mg/dL.

Statistical analysis

Data were analyzed using IBM SPSS Statistics for Windows, Version 21.0 (Released 2012; IBM Corp., Armonk, NY, US). Continuous variables were expressed as mean ± standard deviation (SD). Intergroup comparisons between transudative and exudative effusions were conducted using the Student’s t-test for continuous variables.

Diagnostic performance parameters, including sensitivity, specificity, positive predictive value (PPV), and negative predictive value (NPV), were calculated for each biomarker. Receiver operating characteristic (ROC) curves were generated, and the area under the curve (AUC) was calculated for pleural cholesterol, pleural LDH, and other evaluated parameters. A p-value of < 0.05 was considered statistically significant.

## Results

A total of 80 patients were enrolled in this prospective study, comprising 50 cases of exudative pleural effusion and 30 cases of transudative pleural effusion. The mean pCHOL level was significantly higher in the exudative group (68.5 ± 20.3 mg/dL) compared to the transudative group (25.2 ± 9.6 mg/dL), with p < 0.001. Similarly, both the pleural/serum protein ratio and pleural/serum LDH ratio were markedly elevated in exudates, achieving statistical significance (p < 0.01).

**Figure 2:**
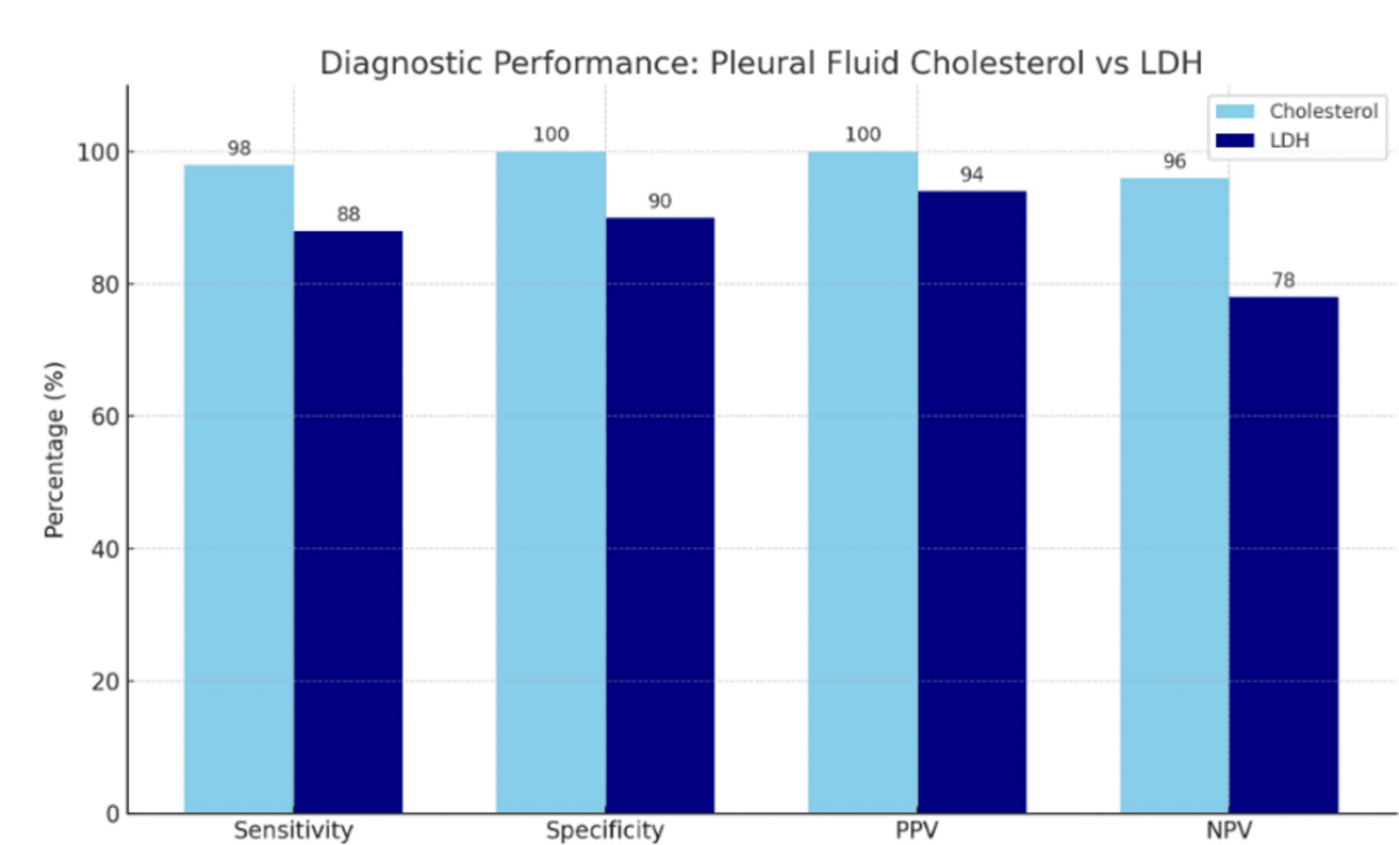
Comparative diagnostic performance of pleural fluid cholesterol and LDH Bar chart illustrating the sensitivity, specificity, positive predictive value (PPV), and negative predictive value (NPV) of pleural fluid cholesterol versus pleural fluid LDH in distinguishing exudative from transudative effusions. Pleural fluid cholesterol demonstrates superior diagnostic metrics across all parameters compared to LDH. Data are represented as percentages (%). Statistical significance was considered at p < 0.05.

Table 1 summarizes the diagnostic performance of pleural fluid biomarkers. Among all evaluated parameters, a pleural cholesterol cutoff > 45 mg/dL demonstrated the highest diagnostic accuracy, with a sensitivity of 98%, specificity of 100%, PPV of 100%, and NPV of 96%. These values exceed those observed with the individual components of Light’s criteria. Specifically, a pleural/serum protein ratio > 0.5 yielded 78% sensitivity and 82% specificity, while a pleural/serum LDH ratio > 0.6 achieved 86% sensitivity and 93% specificity. Additionally, pleural fluid LDH levels exceeding two-thirds of the upper normal serum limit demonstrated 88% sensitivity and 90% specificity. The difference in mean pCHOL levels between exudative and transudative effusions was highly statistically significant (Student’s t-test, p < 0.0001), reinforcing its discriminative power. Figure 2 illustrates the comparative diagnostic performance of pleural cholesterol and pleural LDH, highlighting their respective sensitivity, specificity, PPV, and NPV. Across all diagnostic metrics, pleural fluid cholesterol consistently outperformed LDH, with statistical significance (p < 0.05).

**Table 1:** Diagnostic performance of pleural fluid biomarkers in differentiating exudative from transudative effusions This table summarizes the sensitivity, specificity, positive predictive value (PPV), and negative predictive value (NPV) of the pleural/serum protein ratio, pleural/serum LDH ratio, pleural fluid LDH, and pleural fluid cholesterol in identifying exudative pleural effusions. Pleural cholesterol demonstrated the highest diagnostic accuracy among all parameters evaluated. Data are represented as percentages (%). A p-value of <0.05 was considered statistically significant. LDH: lactate dehydrogenase, ULN: upper limit of normal.

Parameter	Cut-off value	Sensitivity (%)	Specificity (%)	PPV (%)	NPV (%)	
Pleural/serum protein ratio	>0.5	78	82	88	69	
Pleural/serum LDH ratio	>0.6	86	93	95	81	
Pleural fluid LDH	>2/3 ULN	88	90	94	78	
Pleural fluid cholesterol	>45 mg/dL	98	100	100	96	

## Discussion

In this study, pCHOL demonstrated excellent discriminatory power in differentiating exudative from transudative effusions, consistent with previously published literature [13-15]. The comparative analysis of diagnostic parameters highlights the superior performance of pCHOL over traditional Light’s criteria. Across multiple studies, pCHOL has consistently shown higher sensitivity, specificity, PPV, and NPV, reaffirming its clinical utility as a reliable biomarker in pleural fluid analysis.

Unlike Light’s criteria, which may yield false positives in certain clinical scenarios, such as patients with heart failure receiving diuretics or those with parapneumonic effusions, pCHOL retains high diagnostic accuracy, even under such challenging conditions. Notably, combining pCHOL with pleural fluid LDH has been proposed to further enhance diagnostic precision, potentially compensating for borderline values when either marker is used alone. Studies by Yilmaz et al. and Desai and Lee suggest that a combined approach (pCHOL + LDH) can improve both sensitivity and specificity and, in some instances, outperform Light’s criteria [16,17]. In our dataset, however, pCHOL alone demonstrated robust performance at the commonly accepted cutoff of 45 mg/dL, suggesting that it may serve as an effective standalone marker for evaluating pleural effusions. This finding supports its integration into diagnostic workflows, particularly in resource-limited settings where comprehensive biochemical analysis may not be feasible.

The consistent performance of pCHOL across diverse clinical contexts strengthens the argument for its routine inclusion in pleural fluid analysis. Further research involving larger, multi-center cohorts may help determine whether combining pCHOL and LDH provides a significant incremental benefit over using pCHOL alone (Table 2).

**Table 2:** Comparative analysis of sensitivity, specificity, PPV, and NPV of Light’s criteria and pleural fluid cholesterol across different studies This table presents a comparison of diagnostic parameters (sensitivity, specificity, positive predictive value (PPV), and negative predictive value (NPV)) of Light’s criteria versus pleural fluid cholesterol (pCHOL) in differentiating exudative and transudative pleural effusions, as reported in various published studies. The data highlight the consistent diagnostic strength of pCHOL in clinical evaluation.

Parameter		Costa M et al. [13]	Rungta and Jha [14]	Patel and Choudhury [15]
Light’s criteria	Sensitivity	98%	98%	98%
	Specificity	82%	82%	100%
	PPV	–	90%	100%
	NPV	–	82.90%	92%
Pleural fluid cholesterol	Cut-off point	>45 mg/dL	>45 mg/dL	>60 mg/dL
	Sensitivity	90%	90%	98%
	Specificity	100%	99%	100%
	PPV	–	93%	100%

Our primary finding, that a pCHOL cutoff around 45 mg/dL yields approximately 98%-100% sensitivity and specificity, is consistent with multiple prior investigations. For example, Hamal et al. reported mean pCHOL levels of 1.92 mmol/L in exudates and 0.53 mmol/L in transudates, with corresponding sensitivity and specificity of 97.7% and 100%, respectively [7]. Our results closely mirror these values, with near-perfect diagnostic performance.

In contrast, pleural/serum protein and LDH ratios showed more modest diagnostic accuracy. Hamal et al. noted 81% sensitivity and 83% specificity for the protein ratio, and 86% sensitivity with 94.7% specificity for the LDH ratio [7], which align with our own sensitivity figures (78%-86%) (Table 1). Numerous studies have shown that pCHOL misclassifies fewer cases than any individual component of Light’s criteria [14-18].

Several authors have explored cholesterol-based markers versus traditional biochemical criteria. Costa et al. found that combining pCHOL (>45 mg/dL) with LDH (>200 IU/L) yielded 99% sensitivity and 98% specificity, outperforming Light’s criteria (98% sensitivity, 82% specificity) [13]. Similarly, Yilmaz et al. reported that pCHOL alone achieved 95%-96% accuracy, with slight specificity gains when combined with LDH [16].

Our findings support these conclusions: the 45 mg/dL threshold for pCHOL provided optimal diagnostic accuracy, and adding LDH offered minimal additional benefit. Similar outcomes have been reported in studies from Brazil and India. Dhandapani et al. reported 100% sensitivity and specificity with the same cutoff [19], while Rustogi and Gupta observed mean pCHOL values of 70.8 mg/dL in exudates versus 27.8 mg/dL in transudates, with 98.2% sensitivity and 95.7% specificity [20].

These results are corroborated by a recent meta-analysis involving over 3,500 patients across 20 studies, which reported a pooled sensitivity of 88%, specificity of 96%, and AUC of 0.97 for pCHOL [17]. Slightly higher diagnostic figures in single-center studies may reflect variations in patient selection or cutoff thresholds. Nonetheless, the consensus is clear: pCHOL is a highly accurate and reproducible discriminator of pleural effusion type.

Beyond its diagnostic precision, pCHOL offers several practical advantages. Unlike Light’s criteria, it requires only a pleural fluid sample, eliminating the need for concurrent serum testing and thereby simplifying logistics. Costa et al. noted that the combined use of pCHOL and LDH “requires only two laboratory determinations and no simultaneous blood sample,” reducing procedural burden and cost [13]. Rustogi and Gupta likewise described pCHOL as a simple, cost-effective alternative with comparable or better diagnostic performance than Light’s criteria [20].

Additionally, pCHOL is biochemically stable, easily measured with routine automated analyzers, and less affected by factors such as diuretic use, which can confound Light’s criteria by elevating serum-to-effusion protein ratios.

While LDH-based markers remain sensitive, they often lack specificity. In our study, pleural LDH >2/3 ULN correctly identified exudates in 88% of cases, with 90% specificity, similar to previous reports [7,15,16]. However, LDH levels may be elevated in empyema or certain transudates, reducing specificity. In contrast, cholesterol elevation appears more reliably tied to exudative pathophysiology.

Although combining pCHOL with other markers like LDH or protein has been proposed, most evidence, including ours, suggests minimal diagnostic benefit beyond using pCHOL alone. That said, pCHOL should still be interpreted within the broader clinical context. Rare exceptions exist; for instance, cholesterol effusions may occur in benign conditions, and chylous effusions can occasionally elevate cholesterol levels. Nevertheless, compared to traditional markers, cholesterol has demonstrated greater consistency and reliability.

Taken together, our findings, aligned with existing studies and meta-analyses, strongly support pCHOL as a reliable, efficient, and cost-effective marker for differentiating exudative from transudative effusions. Its ease of measurement and robustness across clinical settings make it an excellent candidate for inclusion in routine pleural fluid analysis, especially in resource-constrained environments.

Despite promising results, this study has limitations. First, its single-center design may limit generalizability to broader populations with varied demographic and clinical profiles. Second, the unequal distribution of exudative and transudative effusions, while reflective of real-world patterns, may introduce comparative bias. Third, diagnostic classification relied on Light’s criteria, supported by clinical judgment, but a lack of blinding could have introduced observer bias.

Additionally, we did not control for confounders such as comorbidities (e.g., tuberculosis, malignancy, heart failure) that might influence pleural fluid biochemistry. Variability in laboratory methods across institutions may also affect the reproducibility of pCHOL measurement. Finally, the modest sample size limits the detection of smaller effect sizes. Larger, multicenter studies are needed to validate these findings and further explore the additive value of combining pCHOL with LDH or other markers.

## Conclusions

pCHOL demonstrated superior diagnostic accuracy compared to the individual components of Light’s criteria, achieving nearly 100% sensitivity and specificity in this study. These findings are consistent with prior research and highlight pCHOL as a simple, reliable, and cost-effective biomarker for distinguishing exudative from transudative pleural effusions. Since pCHOL measurement requires only a pleural fluid sample and minimal laboratory infrastructure, it offers a practical alternative in resource-limited settings where full application of Light’s criteria may be constrained. Nonetheless, given the single-center design and modest sample size of this study, larger multicenter investigations are needed to validate these results and to further define the role of pCHOL within routine diagnostic algorithms for pleural effusion.
